# Parathyroid carcinoma as an overlooked etiology of osteoporosis in postmenopausal women: a case report

**DOI:** 10.3389/fendo.2025.1652919

**Published:** 2026-01-23

**Authors:** Jing Su, Shiqiong Lei, Meiyuan Jin, Hongzhi Hu, Wenshan He

**Affiliations:** 1 Department of Breast and Thyroid Surgery, Union Hospital, Tongji Medical College, Huazhong University of Science and Technology, Wuhan, China; 2 Department of Orthopedics, Union Hospital, Tongji Medical College, Huazhong University of Science and Technology, Wuhan, China

**Keywords:** hypercalcemia, hyperparathyroidism, osteoporosis, parathyroid carcinoma, postmenopausal women

## Abstract

Parathyroid carcinoma (PC), an extremely rare endocrine malignancy, disrupts calciumphosphorus homeostasis and lead to musculoskeletal system disorders including osteoporosis, bone pain and pathological fractures. For postmenopausal women, osteoporosis is a common disease. Therefore, secondary osteoporosis is often overlooked in this demographic. We report a 54-year-old woman presenting to Orthopedics Department due to arthralgia diagnosed with severe postmenopausal osteoporosis (PMO). After pharmacotherapy, the patient’s symptoms did not show significant improvement. Subsequent endocrine evaluation revealed hyperparathyroidism as the underlying cause. Following parathyroidectomy, histopathological evaluation confirmed the diagnosis of PC and her osteoporosis symptoms improved. This case highlights the critical need for postmenopausal women with osteoporosis to determine whether their condition is primary or secondary in nature. Moreover, the therapeutic principles for managing primary and secondary osteoporosis differ substantially. Early etiological identification is essential to optimize management.

## 1 Introduction

Parathyroid carcinoma, an extremely rare endocrine malignancy, comprises less than 1% of primary hyperparathyroidism (PHPT) cases and less than 0.005% of all cancers (1–3). The median onset age is 44–65 years with no gender prevalence (4, 5). It more manifests as a sporadic disease, occasionally being a part of specific syndromic and non-syndromic endocrine diseases (6). The overexpression of *CCND1* and *EZH2*, mutations in *CDC73/HRPT2*, and promoter methylation of *APC*, *SFRP1*, *SFRP2*, *SFRP4*, *CDKN2A* and *WT1* are implicated in parathyroid tumorigenesis (7). Whereas, the etiology and pathogenesis are still incompletely clear. Pre-operative diagnosis remains challenging due to nonspecific biomarkers. Almost all PCs are functional tumors, causing hypercalcemia-related renal, bone, digestive and psychiatric manifestations (5, 8). Among these, bone manifestations, including osteoporosis, bone pain and pathological fractures, are predominant.

Osteoporosis, a metabolic skeletal disorder characterized by low bone mass and degeneration of bone tissue structure, affects 200 million people worldwide (9). It can be categorized into primary osteoporosis (including PMO and senile osteoporosis) and secondary osteoporosis. The diagnostic criteria for PMO is the occurrence of fractures or dual-energy X-ray (DXA) T-score ≤ -2.5 standard deviations (SDs) at the femoral neck, total hip or lumbar spine (10). However, there is currently no consensus regarding specific evaluations to exclude secondary osteoporosis. Especially for postmenopausal women affected by estrogen, secondary osteoporosis is often overlooked.

This study is aimed to report a case of parathyroid carcinoma due to osteoporosis missed in postmenopausal women with arthralgia, which can be a warning to orthopedic surgeons.

## 2 Case report

**Figure 1:**
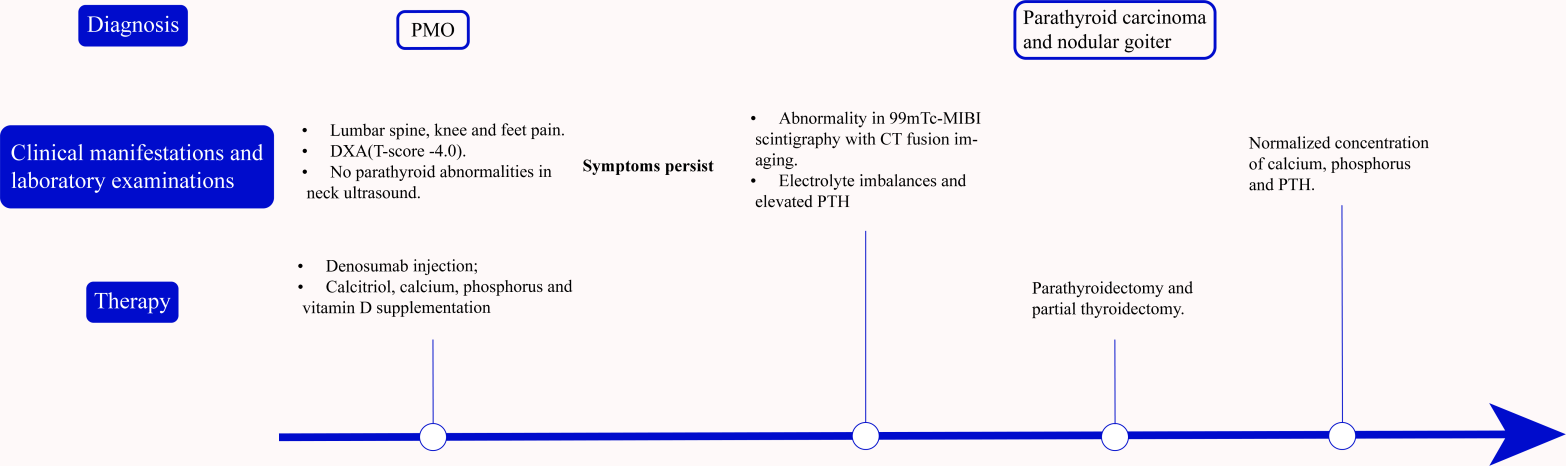
A timeline illustrating the progression from initial mismanagement of osteoporosis to definitive surgical cure of parathyroid carcinoma.

**Figure 1** maps the clinical timeline beginning with the orthopedic consultation of a 54-year-old postmenopausal patient with a history of hypertension, coronary artery disease, and chronic hepatitis B, without prior fragility fractures or known hereditary disorders. She presented to the Orthopedics Department in July 2024 with lumbar spine, knee and feet pain. A comprehensive physical examination was unremarkable. X-ray and DXA (T-score -4.0) confirmed severe osteoporosis. Combining the age, sex and the examination results, the patient was diagnosed with severe PMO, receiving denosumab injection and supplementation of calcitriol, calcium, phosphorus and vitamin D. At this time, neck ultrasound showed no parathyroid abnormalities and laboratory evaluation for secondary osteoporosis was not performed.

**Figure 2:**
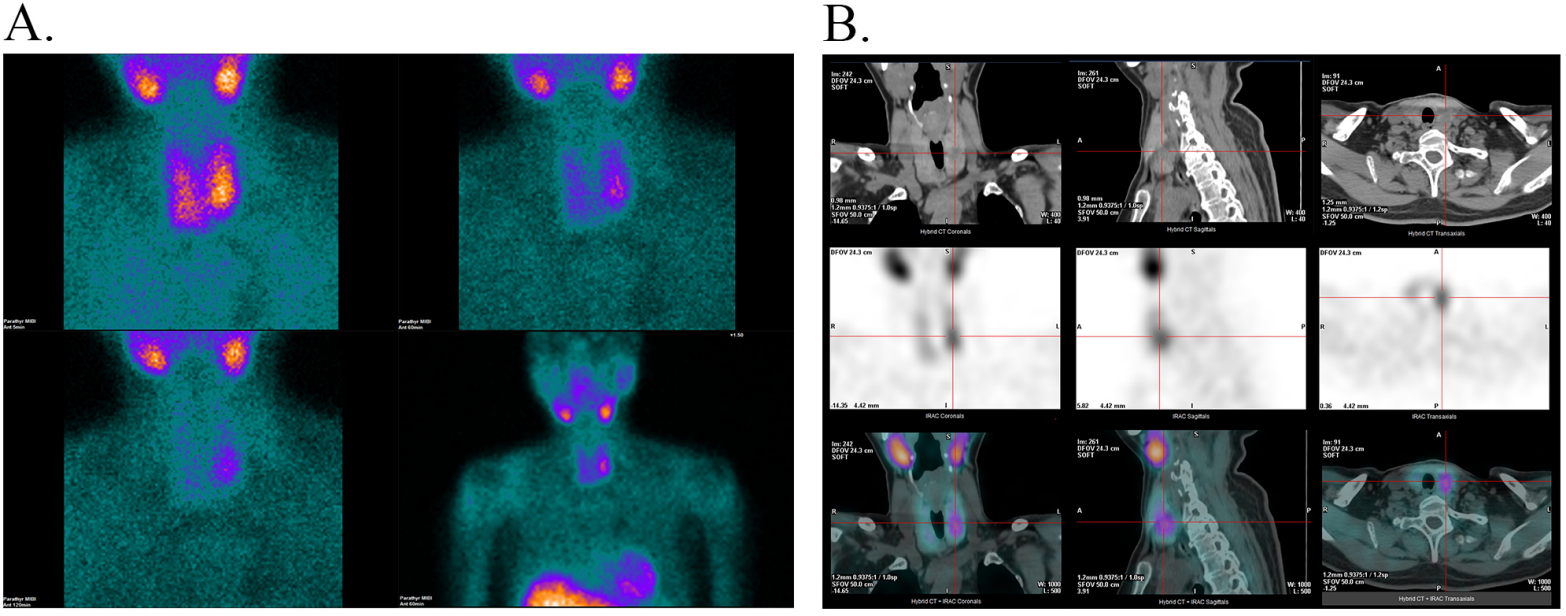
**(A)** 5 minutes, 1 hour and 2 hours after ^99m^Tc-MIBI injection. **(B)** The ^99m^Tc-MIBI scintigraphy with CT fusion imaging findings.

Two months later, due to persistent symptoms, the patient was referred to the endocrinology department. In view of the patient’s prior abnormal laboratory results, including hypercalcemia (3.28 mmol/L; normal, 2.03–2.54 mmol/L), hypophosphatemia (0.72 mmol/L; normal, 0.96–1.62 mmol/L), and elevated alkaline phosphatase (ALP, 389 U/L; normal, 40–150 U/L), ^99m^Tc-sestamibi (MIBI) scintigraphy with computed tomography (CT) fusion imaging was performed to investigate potential parathyroid issues. Early-phase imaging (5 minutes post-injection) showed normal thyroid radiotracer uptake, whereas significantly increased radioactivity in the left inferior-middle thyroid lobe. Delayed imaging (1- and 2-hours post-injection) demonstrated persistent but lower activity in the left inferior-middle thyroid lobe (**Figure 2**). CT fusion localized a lesion (1.7 × 1.8 × 2.4 cm) posterior to the inferior-middle left thyroid lobe, but its origin remained indeterminate (**Figure 2**).

The patient was subsequently hospitalized. A thyroid ultrasonography revealed bilateral thyroid enlargement with multiple nodules and a solid hypoechoic nodule (27.9 × 17.1 × 13.6 mm) located dorsally in the inferomedial left thyroid lobe.

Preoperative laboratory findings showed normal thyroid function, increased serum calcium levels of 2.70 mmol/L (normal, 2.03-2.54 mmol/L), increased plasma parathyroid hormone (PTH) levels over 3000 pg/ml (normal, 15.0-68.3 pg/ml) and a low serum phosphorus level of 0.51 mmol/L (normal, 0.96-1.62 mmol/L).

## 3 Diagnostic assessment, treatment, and outcomes

The biochemical triad of hypercalcemia with hypophosphatemia and markedly elevated PTH supported PHPT. ^99m^Tc-MIBI/CT fusion imaging and neck ultrasonography to the posterior inferomedial left thyroid lobe increased the post-test probability. Key diagnostic challenges included anchoring on primary postmenopausal osteoporosis and the limited performance of parathyroid ultrasonography in the setting of multinodular thyroid disease. The differential encompassed primary osteoporosis and non–PTH-mediated causes of hypercalcemia. Within primary hyperparathyroidism, adenoma, hyperplasia, and carcinoma were considered. Histopathology served as the reference standard.

Guided by preoperative localization, the left superior parathyroid gland was preserved *in situ* and the left inferior parathyroid gland was excised together with ipsilateral hemithyroidectomy and isthmusectomy. An intraoperative frozen section confirmed parathyroid neoplasm, and the definitive diagnosis of parathyroid carcinoma with coexistent nodular goiter was established on permanent histopathology.

On the second postoperative day, laboratory tests showed serum calcium 2.23 mmol/L (normal, 2.10–2.55 mmol/L), serum phosphate 0.93 mmol/L (normal, 0.81–1.45 mmol/L), and plasma PTH 38.7 pg/mL (normal, 15.0–68.3 pg/mL), indicating an adequate initial biochemical response. Adjuvant radiotherapy was administered after surgery. On serial follow-up, neck ultrasonography showed no new or suspicious abnormalities. Intermittent biochemical fluctuations of plasma PTH and of serum calcium and phosphate were observed. However, deviations from the reference ranges were small and not sustained, and the patient had no hypercalcemic symptoms or related complications. At the last follow-up, the patient remained clinically well with stable biochemistry. Longitudinal surveillance of serum calcium and parathyroid hormone is planned to monitor for recurrence.

## 4 Discussion

Osteoporosis is associated with aging and menopause, affecting women more than men in those over fifty (11). The postmenopausal decline in estrogen levels contributes to decreased bone mineral density and increased musculoskeletal pain (12). As PMO is the primary contributor to osteoporosis, orthopedic doctors frequently attribute musculoskeletal pain in postmenopausal women to PMO without considering other diagnoses. Current clinical practice guidelines recommend assessment for secondary causes of osteoporosis. However, there is no universally accepted laboratory testing standard, and the extent of testing is guided by the patient’s history and clinical findings. But serum calcium, serum phosphorus, alkaline phosphatase (ALP), creatinine and albumin are fundamental examinations (10). If the results of the aforementioned laboratory data are abnormal, further examinations are required. In our case, the normal parathyroid ultrasound result may divert clinical attention from potential parathyroid-related issues.

The parathyroid glands exhibit variable anatomical positions, often adjacent to the thyroid, which limits ultrasonographic differentiation of parathyroid pathologies in patients with concurrent thyroid nodules. Preoperative ultrasound demonstrates a sensitivity of 71%-93% in detecting parathyroid abnormalities (13, 14). In our case, since May 2022, serial neck ultrasounds annually have documented progressive enlargement and multiplication of thyroid nodules without detectable parathyroid abnormalities before October 2024. For doctors who are not thyroid surgeons or endocrinologists, normal ultrasound findings should not preclude parathyroid dysfunction. PTH evaluation remains essential in conjunction with abnormal electrolyte level. Taken together, in this patient the coherent sequence of lesion localization on multimodal imaging, followed by rapid postoperative biochemical normalization with symptomatic improvement, supports the internal validity of our inference that hyperparathyroidism was an important contributor, among multiple etiologies, to the skeletal manifestations.

The pre-operative diagnosis of PC remains challenging due to nonspecific biochemical and clinical manifestations. Comparing with benign parathyroid adenomas (BPA), the level of calcium, PTH and ALP in PC are markedly higher. A cohort study from South Korea showed that an ALP level of 285 IU/L had predictive value for PC (sensitivity 83%, specificity 97%) (15). In our case, initial ALP elevation (338U/L; normal, 40-150U/L) was documented on May 1, 2023, followed by hypercalcemia and hypophosphatemia detection on February 6, 2024. The absence of PTH measurement at the initial stage precludes definitive assessment and likely contributed to the delayed diagnosis. Previous studies have reported an association between hyperparathyroidism and thyroid disease, and lower PTH levels have been considered a potential risk factor for thyroid cancer (16, 17). However, the association between PTH and lesion size differs. Lower PTH has been linked to larger tumor foci and multifocality in thyroid cancer, while higher PTH has been associated with larger nodule size in thyroid nodules (16–18). Overall, evidence is limited and mainly comes from retrospective or prospective observational studies, and the mechanisms remain unclear. These observations raise two critical questions: 1) whether PC was already present in 2023, and 2) whether thyroid nodules progression reflects PTH-mediated effects. It is noteworthy that, PHPT patients with prior thyroid disease tend to be more asymptomatic compared to those without (19). This may explain the patient’s delayed orthopedic visit.

In our case, osteoporosis cannot be attributed to a single etiology. In a midlife woman, postmenopausal bone loss plausibly coexisted with PTH-mediated skeletal effects. Although parathyroidectomy normalized calcium, phosphate, and PTH, the patient remained osteoporotic and required ongoing anti-osteoporosis therapy. Interval DXA was not yet available due to short postoperative follow up. These findings support a combined contribution of postmenopausal osteoporosis and secondary osteoporosis due to hyperparathyroidism. At the level of an individual patient, there is no reliable method to quantify the relative weight of these mechanisms. Clinically, secondary causes should be systematically evaluated. When identified, these causes should be prioritized for treatment because management strategies differ substantially between primary and secondary osteoporosis.

Until now, surgery is still the gold standard of the treatment for PC. As for patients with unresectable PC, medication is mainly aimed to manage PTH-induced hypercalcemia. To be mentioned, pharmacological management differs fundamentally between PTH-mediated hypercalcemia and primary osteoporosis, despite partial overlap in drug classes (**Table 1**). In hypercalcemia driven by hyperparathyroidism, medications are primarily used for rapid calcium lowering and short-term suppression of osteoclastic bone resorption, often at higher doses and shorter dosing intervals than those used for fracture prevention in primary osteoporosis. By contrast, primary osteoporosis treatment aims at long-term fracture-risk reduction with lower-dose, intermittent regimens of antiresorptives, while anabolic agents (PTH analogues) are contraindicated in the setting of hypercalcemia or osteoporosis secondary to hyperparathyroidism. These differences underscore the clinical importance of excluding secondary causes before labeling postmenopausal women as having primary osteoporosis and initiating routine anti-osteoporotic therapy (20–24).

**Table 1:** Summary of medications in PTH-induced hypercalcemia and primary osteoporosis (20, 21, 23–27).

Medications	PTH-induced hypercalcemia	Primary osteoporosis
Bisphosphonates	Indicated.	Indicated.
	1. Pamidronate: 60-90mg IV^a^ (over 2–24 hours), can be repeated every 2–3 weeks. 2. Zoledronic: 3-4mg IV (over 15–30 minutes), can be repeated in 7 days (if target calcium level is not achieved) and then every 3–4 weeks as needed. 3. Ibandronate: 2-4mg IV.	1. Alendronate: 70mg PO^b^ once weekly or 10mg PO once daily. 2. Zoledronic: 5mg IV once yearly. 3. Risedronate: 35mg PO once weekly or 5mg PO once daily or 150mg PO once monthly. 4. Ibandronate: 150mg PO once monthly or 2-3mg IV every 3 months. 5. Minodronic acid: 1mg PO once daily.
Denosumab (RANKL inhibitor)	Indicated. 120 mg SC^c^, repeat at weeks 1, 2 and 4, then monthly thereafter. (FDA has approved denosumab for osteoporosis and malignancy-related hypercalcemia, but it is not included in domestic indications.)	Indicated. 60mg SC every 6 months.
Calcitonin	Indicated.	Indicated.
	Salmon calcitonin: 4–8 IU/kg IM^d^ or SC every 6–12 hours for 48–72 hours.	1. Elcatonin: 20 U IM once weekly or 10U IM twice weekly. 2. Salmon calcitonin: 100 IU SC/IM every other day or 200 IU intranasally once daily.
Estrogen	Not indicated.	Indicated. An individualized treatment regimen is recommended.
SERMs^e^	Not indicated.	Indicated. Raloxifene: 60mg PO once daily.
Conjugated estrogens/bazedoxifene	Not indicated.	0.45 mg/20 mg PO once daily.
PTHa^f^	Contraindicated.	Indicated. (Contraindications: hypercalcemia and osteoporosis induced by hyperparathyroidism.) 1. Teriparatide: 20μg SC once daily. 2. Abaloparatide: 80μg SC once daily.
Romosozumab	Not indicated.	Indicated. (Currently in the clinical trial phase in China.) 210mg SC once monthly for 12 months.
Vitamin D analogue	Avoid/Generally contraindicated.	Indicated. (Contraindications: hypercalcemia.)
Vitamin K analogue	Not indicated.	Indicated. Menatetrenone: 15mg PO three times daily.
Cinacalcet	Indicated. 30 mg PO twice daily, increasing dose incrementally.	Not indicated.

a IV, intravenous.

b PO, orally.

c SC, subcutaneous.

d IM, intramuscular.

e SERMs, selective estrogen receptor modulators.

f PTHa, parathyroid hormone analogue.

## 5 Patient perspective

Following diagnosis, the patient’s main concerns were the rarity of parathyroid carcinoma, the lack of standardized therapeutic guidelines, and uncertainty regarding prognosis.

## Data Availability

The original contributions presented in the study are included in the article/supplementary material. Further inquiries can be directed to the corresponding authors.
